# Influence of Thermo-Oxidative Ageing on the Thermal and Dynamical Mechanical Properties of Long Glass Fibre-Reinforced Poly(Butylene Terephthalate) Composites Filled with DOPO

**DOI:** 10.3390/ma10050500

**Published:** 2017-05-04

**Authors:** Daohai Zhang, Min He, Weidi He, Ying Zhou, Shuhao Qin, Jie Yu

**Affiliations:** 1 Department of Polymer Material and Engineering, College of Materials and Metallurgy, Guizhou University, Guiyang 550025, China; zhangdaohai6235@163.com (D.Z.); hemin.851@163.com (M.H.); 2 National Engineering Research Center for Compounding and Modification of Polymeric Materials, Guiyang 550014, China; demon3301932@sina.com (W.H.); zhouying_0304@126.com (Y.Z.)

**Keywords:** long glass fibre-reinforced poly(butylene terephthalate), DOPO, thermo-oxidative ageing, dynamic mechanical properties, composites

## Abstract

In this work, the long glass fibre-reinforced poly(butylene terephthalate) (PBT) composites filled with 9,10-dihydro-9-oxa-10-phosphaphenanthrene-10-oxide (DOPO) were prepared by melt blending, and the influence of thermo-oxidative ageing on the static and dynamic mechanical properties, thermal behaviours and morphology of composites with different ageing time at 120 °C were investigated and analysed. The results showed that the mechanical properties decreased in the primary stage of ageing, while embrittlement occurs in the later period, and the crystallinity of PBT decreases first, and then recovers to some extent. The scanning electron microscopy (SEM) photos of the samples indicated that the obvious crack appeared on the sample surface and a deeper, broader crack occurred with a longer ageing time. The results of energy dispersive X-ray analysis (EDAX) proved the DOPO filler diffused to the sample surface by measuring the content of phosphorus. Thermal gravimetric analysis (TGA) curves showed that the thermal stabilities of composites increased with longer ageing time, as did the values of the limited oxygen index (LOI). Meanwhile, the results of dynamic mechanical analysis (DMA) indicated that the glass transition temperature shifted to a higher temperature after ageing due to the effect of crosslinking, and both the crosslinking and degradation of PBT molecular chains act as the main factors in the whole process of thermo-oxidative ageing.

## 1. Introduction

Poly(butylene terephthalate) (PBT) is a type of typical semi-crystalline polymer material with wide applications due to its properties, such as rigidity, a high rate of crystallization, and hardness [1,2]. However, PBT has a relatively low mechanical strength, especially impact strength, which shows a sensitive effect to notches [3,4]. In recent years, there have been growing interest in long glass fibre (LGF)-reinforced polymer composites because of their light weight, high mechanical strength, and low cost, which have been applied in automobiles, construction, and aviation [5,6,7]. With these advantages, the LGF-reinforced PBT composites can become more suitable for highly demanding situations than original PBT materials.

To improve the flame retardant properties of polymer composites, flame retardant filling in composites has been known as a promising method. Traditional halogen flame retardants can act to effectively function in flame retardant polymeric materials with a relatively low volume of addition, but its application is limited due to the toxic combustion products [8,9,10]. With the progress of the phosphorus chemical industry, 9,10-dihydro-9-oxa-10-phosphaphenanthrene-10-oxide (DOPO) and its derivatives, as a novel type of phosphorus-containing flame retardant, has received remarkable attention due to the high reactivates [11]. DOPO is considered to be an environmentally-friendly flame retardant additive, which has many advantages, such as low smoke, no halogen, excellent thermal stability, and excellent flame retardant performance [12,13,14], and its applications in polymer composites are increasingly significant.

In addition, some properties of the polymeric materials will degrade, such as yellowing, losing lustre, or a significant decline in the mechanical properties when exposed to water, ultraviolet radiation, or harsh environments during their processing, storage, and service life [15]. These phenomena are called the ageing of the polymer materials, which will lead to limitations in many fields of application. Among the environmental elements, heat and oxygen can usually act on materials at the same time, especially for polymers which, with relatively low heat resistance, are easily oxidated and degraded. In order to clarify and overcome these disadvantages, it will be of great importance to investigate the influences on the properties of polymer composites in thermo-oxidative ageing.

In this article, the LGF-reinforced PBT composites filled with DOPO (PBT/LGF/DOPO) are prepared by the melt-impregnation process. The static and dynamic mechanical properties, thermal properties, flammability, and morphologies of composites with different thermo-oxidative ageing times are investigated in detail. The aim of this work is to evaluate the influence and mechanism of thermo-oxidative ageing on PBT/LGF/DOPO composites and to investigate the feasibility to improve the durability of the LGF-reinforced PBT flame retardant composites.

## 2. Experimental

### 2.1. Materials

Poly(butylene terephthalate) (PBT) (grade L1082) was acquired by Sinopec Yizheng Chemical Co., Ltd. (Yizheng, China). The continuous glass fibre (grade 988), treated with a silane coupling agent in this study, was produced by JU SHI Limit Co. (Hailing, China). The diameter of the glass fibre was 10 μm. The flexibilizer was supplied by USA Lubrizol Advanced Materials, Inc. (Wickliffe, OH, USA) DOPO was obtained from Chinese Wansheng technology Co., Ltd. (Linhai, China).

### 2.2. Preparation of the Samples

PBT/DOPO master batches were prepared by mixing DOPO and PBT (*w*(DOPO):*w*(PBT) = 1:1) in a two-screw extruder (TSE–40A, L/D = 40, D = 40 mm, Coperion Keya machinery, Co., Ltd., Nanjing, China) at 210–235 °C. The master batches were dried for six hours at 80 °C to remove water rmoisture to ensure that the moisture content of the master batches was under 0.01% before use (measured by a ZS-201B plastic moisture tester, Susai Co., Ltd, Shenzhen, China).

**Figure 1:**
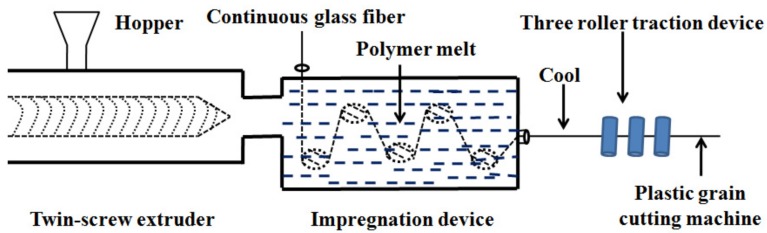
The melt-impregnation process of the LGF(40%)/PBT composites.

Then, the PBT/LGF master batches and 15% thermoplastic polyurethane as a flexibilizer were extruded (type TSE-40A/400-44-22, L/D = 40, D = 40 mm, Coperion Keya Machinery Co., Ltd., Nanjing, China). The temperatures from the hopper to the die at six different zones were 210 °C, 215 °C, 220 °C, 225 °C, 230 °C, and 235 °C, respectively; the screw speed was 200 rpm and the impregnation temperature was 260 °C. The continuous strand was then cut into pellets 11 mm in length for injection moulding. The melt-impregnation process of the LGF/PBT composites is displayed in Figure 1.

Then the PBT/DOPO masterbatches were blended with the PBT/LGF (40 wt %) master batches and additional PBT matrix to keep the content of the glass fibre at 20 wt % and DOPO at 9 wt % in the PBT/LGF/DOPO composites. Blended composites were injection moulded (type CJ80M3V, Chen De Plastics Machinery Co., Ltd., Chengde, China) at 235 °C into various specimens for testing and characterization.

The thermo-oxidative ageing course then proceeded by the oven method. PBT/LGF/DOPO specimens were put into an air-blowing thermostatic oven at 120 °C. The ageing lasted 60 days, and every 10 days several specimens were taken out for testing.

### 2.3. Measurements and Characterization

#### 2.3.1. Mechanical Property Test

The tensile and bending strengths of samples were carried out on a tensile tester (type WDW-10C, Shanghai Hualong Testing Instrument Co., Ltd., Shanghai, China) with a crosshead speed of 100 mm/min (GB/T 1040.1-2008). Notched Izod impact strength was measured using a pendulum impact testing machine (type ZBC-4B, Xinsansi Measurement Technology Co., Ltd., Shenzhen, China). The radius of the notch used in the specimens was 2 mm (GB 1043-79). All of the tests were performed at 25 ± 2 °C. The results were the average values of at least five specimens.

#### 2.3.2. Differential Scanning Calorimetry (DSC)

The DSC thermograms were carried out under nitrogen using a TA instrument Q10 for measuring non-isothermal crystallization and melting behaviours of the composites (ASTM D3418). Thermograms were recorded at a heating or cooling rate of 10 °C/min in the second cycle from 40 °C to 270 °C. The percent crystallinity was determined by dividing the heat of fusion value by 145 J/g and the heat of fusion of 100% crystalline PBT as per the equation:

(1) $$X_{C}=\frac{\Delta H_{m}}{(1-X)\Delta H_{m}^{0}}$$

where $\Delta H_{m}$ is crystallization enthalpy of the samples (J/g), $\Delta H_{m}^{0}$ is the enthalpy value of the melting of a 100% crystalline form of PBT, and X is the weight fraction of the filler.

#### 2.3.3. Morphology Observation

Scanning electron microscopy (SEM) images were obtained on a FEI Quanta 250 (FEI Co., Ltd., Hillsboro, OR, USA) to investigate the surface morphology and impact fracture of the samples. SEM graphs of the composites were recorded after gold coating surface treatment, with an accelerating voltage of 25 kV.

#### 2.3.4. Energy Disperse X-ray Analysis (EDAX)

EDAX was applied to analyse the element content in an area of the sample surface (INCA-350X, Oxford Instruments, Oxford, UK).

#### 2.3.5. Limiting Oxygen Index (LOI)

The LOI value was obtained by using an LOI instrument (type JF-3, Jiangning Analysis Instrument Factory, Nanjing, China) on sheets 120 mm × 6.5 mm × 3 mm according to the standard oxygen index test ASTM D2863-77. The LOI value is calculated according:

LOI = [O_2_]/([O_2_] + [N_2_]) × 100%

where [O_2_] and [N_2_] are the concentration of O_2_ and N_2_, respectively.

#### 2.3.6. Thermal Gravimetric Analysis (TGA)

Thermal gravimetric analysis (TGA) of samples was examined under nitrogen flow with a flow rate of 40 mL/min in a temperature range of ambient to 700 °C, at a heating rate of 20 °C/min, by a TA Instrument Q-50 thermo-gravimetric analyser. Approximately 8-10 mg specimens were used in this test.

#### 2.3.7. Dynamic Mechanical Analysis (DMA)

The dynamic mechanical properties were studied with a Q800 Metravib RDS viscoanalyser at a heating rate of 2 °C/min over a temperature range from −60 to 120 °C. The low-temperature measurements were performed in a stream of dry air cooled with liquid N_2_, and the high-temperature measurements were carried out in a stream of dry N_2_. The samples were tested with an imposed frequency of 5 Hz and an oscillation amplitude of 10 μm in the bending mode.

## 3. Results and Discussion

### 3.1. Mechanical and Crystallization Properties

**Figure 2:**
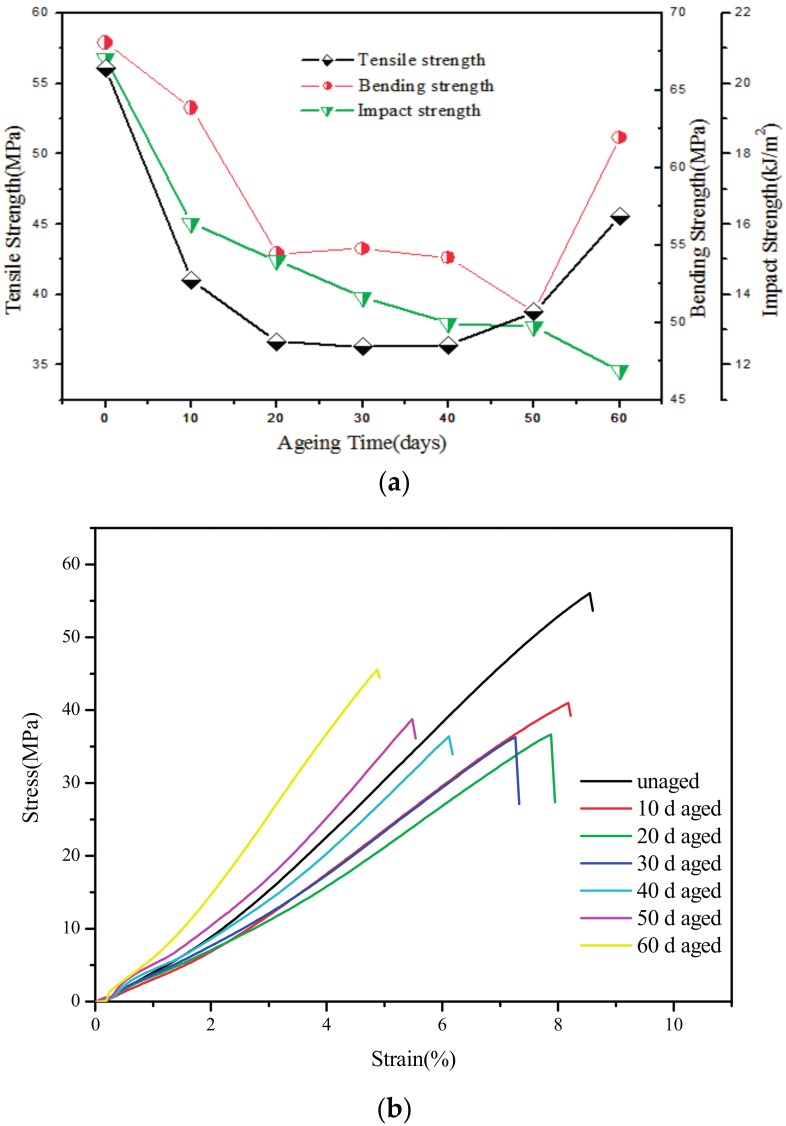
(**a**) Mechanical properties and (**b**) tensile stress-strain curve of PBT/LGF/DOPO composites with different ageing times.

The variation of the mechanical properties and the tensile stress-strain curve of PBT/LGF/DOPO composites with different ageing times are displayed in Figure 2. From Figure 2, the tensile strength and flexural strength of PBT/LGF/DOPO composites, firstly, decrease and then increase with increasing ageing time. Form Figure 2, the tensile stress-strain curve of PBT/LGF/DOPO composites show a brittle fracture. Additionally, the elongation at break of PBT/LGF/DOPO composites gradually decreases with increasing ageing time. The tensile strength of PBT/LGF/DOPO composites (20 days) is decreased by 38.24%, compared with that of unaged specimens. However, flexural strength of PBT/LGF/DOPO composites (50 days) is decreased by 24.19%, compared with that of unaged specimens. While the impact strength of samples decreases in the whole ageing period. The notched izod impact strength of PBT/LGF/DOPO composites (60 days) is decreased by 43.56%, compared with that of unaged specimens. These results indicate that the mechanical properties decrease obviously in the early period of ageing by the effect of heat flow and oxidation. In the later period of ageing (50–60 days), the increase of the tensile strength and the continual decrease of the impact strength indicate that the composites become hard and brittle, which means embrittlement of material occurs. It has been reported that long glass fibre reinforcement other kinds of matrix, such as polypropylene and polyamide 6, in thermo-oxidative ageing both show a global decrease in mechanical strength, without recovery or post-cure [8,16]. Compared with those composites, the performance of PBT/LGF/DOPO composites shows a distinct difference in the ageing process.

**Figure 3:**
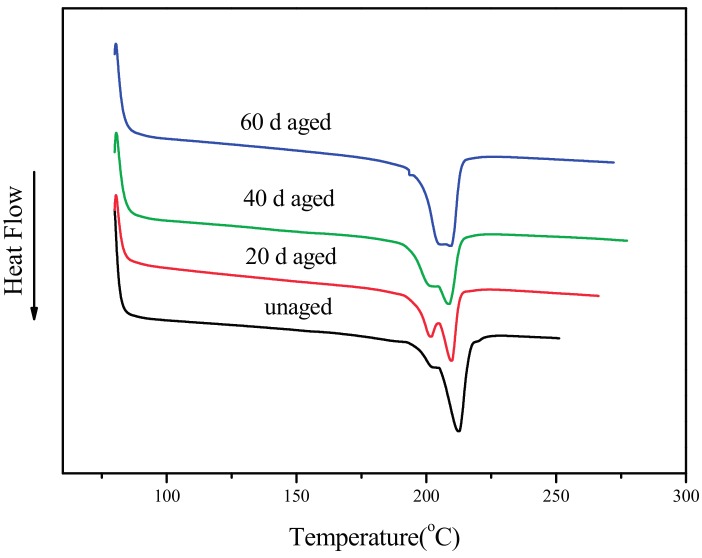
DSC melting curves of PBT/LGF/DOPO composites with different ageing times.

To analyse the variation of the mechanical properties, DSC is conducted to provide the evidence of the variations of the mechanical properties. The melting curves of samples on the second cycle with different ageing times are shown in Figure 3, and the crystallinity (X_c_), melting enthalpy (ΔH_m_), and melting temperature (T_m_) of each sample obtained from the curves and melting parameters are listed in Table 1. The values of X_c_ decrease from 24.13% to 21.44% in the first 20 days, then increases to 23.24% after 60 days of ageing, with the same trend of the variation of tensile and bending strengths stated above. The decrease in X_c_ in the early period of ageing can be attributed to the cross-linking of the PBT matrix which occurs in the amorphous region with the effect of heat and oxygen [17]; for this reason they also show lower melting points [18]. Though the ageing temperature is much lower than the melting temperature of PBT and the mobility of PBT molecular chains is limited, the chains can still relax and rearrange in a relatively long time (at least 10 days) according to the time-temperature equivalence principle. The cross-linking leads the variation which can cause the amorphous region to enlarge, while the crystalline region diminishes to make X_c_ decrease. With longer ageing time (over 40 days), the degradation and oxidation of PBT caused by thermo-oxidative ageing becomes outstanding. Free radicals and peroxides are generated in the PBT matrix due to the thermo-oxidative ageing, which can accelerate the molecular chains to rupture and damage the matrix. The P–H bond in the DOPO filler can react with the hydroxide radicals to dehydrate PBT and form some partial carbonized matrix. This partially-carbonized matrix can lead to the embrittlement of composites. With both of the effects in the later period of ageing, the X_c_ increases slightly, then remains stable, as does the variation trend of the increase of tensile strength and the decrease in impact strength.

It is observed that the peaks change in the melting curves with different ageing times. It has been reported that PBT has a stage of multiple melting in the melting period [19,20,21]. In this work, unaged, 20 d, and 40 d aged composites have two obvious melting peaks, while that with 60 d aged composite has just one peak. This can be attributed to the structure reforms of PBT molecules in thermal ageing: after 60 days of ageing at 120 °C, the ageing time is long enough to be equal to a heat treatment of a higher temperature. The peaks of lower temperature in the melting curve reflect the difficulty of molecule movement. With a long enough time at this temperature, the peaks move to high temperatures gradually so that there is only one peak observed. This kind of heat treatment makes the X_c_ increase, which can be reflected by the post-cure (the increase in composites hardening over time) and embrittlement of composites in the later period of ageing.

### 3.2. Morphology Observation

**Figure 4:**
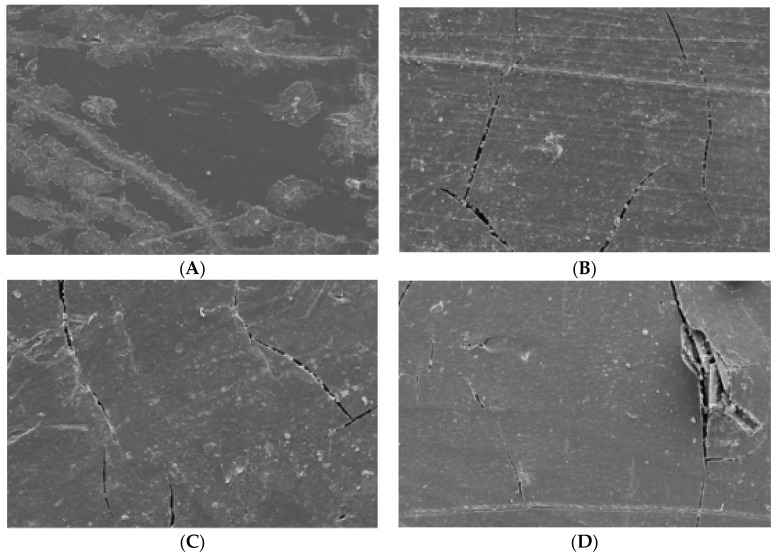
Effect of thermo-oxidative aging on morphology (500×) of PBT/LGF/DOPO composites surface: (**A**) unaged; (**B**) 20 d aged; (**C**) 40 d aged; and (**D**) 60 d aged.

It has been indicated that there are two methods of material failure: one is fracture from the rim of materials which presents a regular comb’s teeth appearance; the other one is fracture from a point inside the material which diverges to a dendritic appearance [8]. Surface morphologies of PBT/LGF/DOPO composites analysed by SEM are shown in Figure 4. Compared with unaged samples, cracks appear on all aged sample surfaces to different extents, and with longer ageing time the cracks become deeper, longer, and broader. This is attributed to the difference of thermal and physical properties between the glass fibres and the PBT matrix, which makes the heat absorption and transmission uneven in PBT/LGF composites. The internal stress created in the procedure of heating and cooling causes this debonding and cracking. The performances of cracks on the composite surface indicate that the heat damage and molecular chain breaking start from the surface and move toward the inner structure, which can cause a decrease in the mechanical properties as stated above.

**Figure 5:**
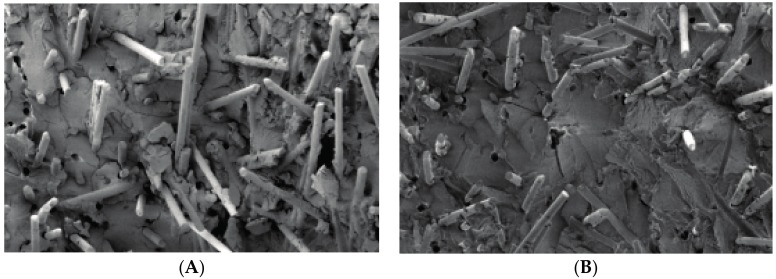

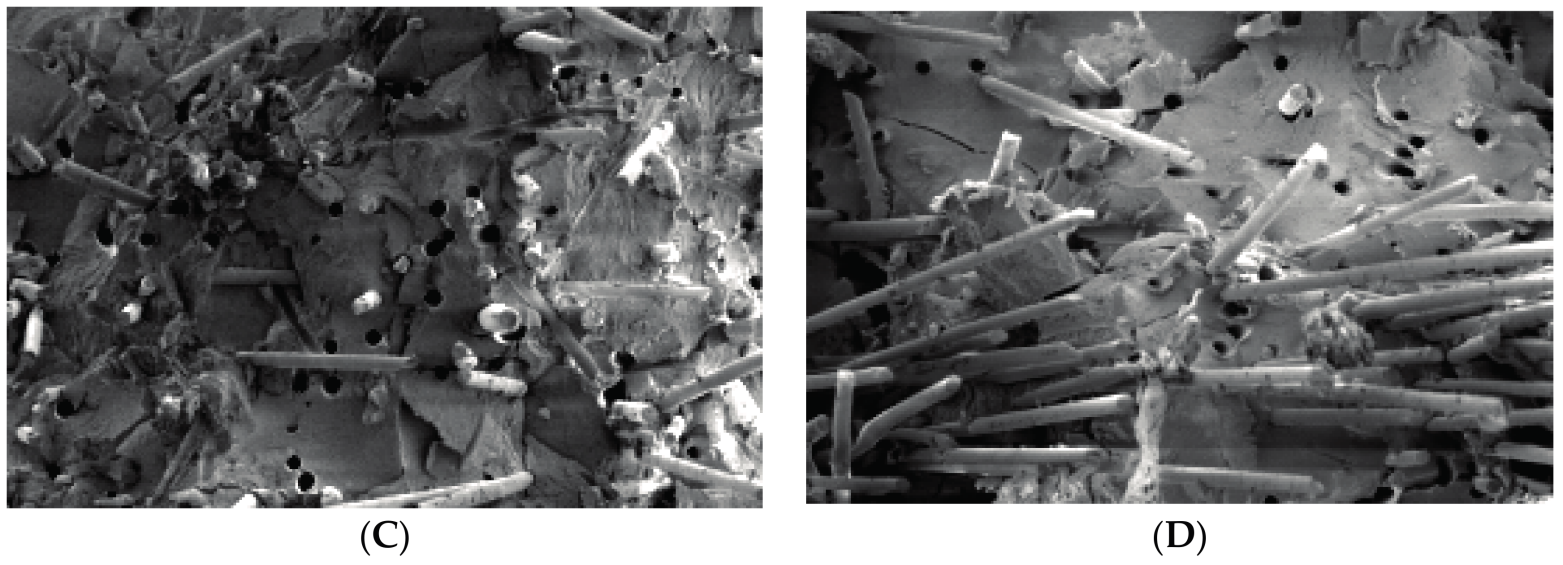
Effect of thermo-oxidative aging on morphology (500×) of PBT/LGF/DOPO composites impact fracture: (**A**) unaged; (**B**) 20 d aged; (**C**) 40 d aged; and (**D**) 60 d aged.

For fibre-reinforced polymer composite deformation, there are three methods of energy absorption: the breakage of the glass fibres, fibres pulling out, and polymer matrix rupture [9]. When stress acts on composites, the internal shear stress will transfer the external stress from the matrix to the interface with the fibre [10]. Figure 5 displays the morphology of impact fractures of samples with 0–60 d ageing. For unaged samples shown in Figure 5A, a few glass fibres are pulled out and also show a rough surface coated with PBT matrix, which means the interface between the glass fibres and matrix are still firm. For aged samples, as shown in Figure 5B–D, more glass fibres are pulled out with a smooth surface, and more holes can be observed with longer ageing time, which infers that the interface debond severely after ageing. The interface debonding also leads to the global decrease of impact strength in the whole ageing period.

### 3.3. EDAX Results

**Figure 6:**
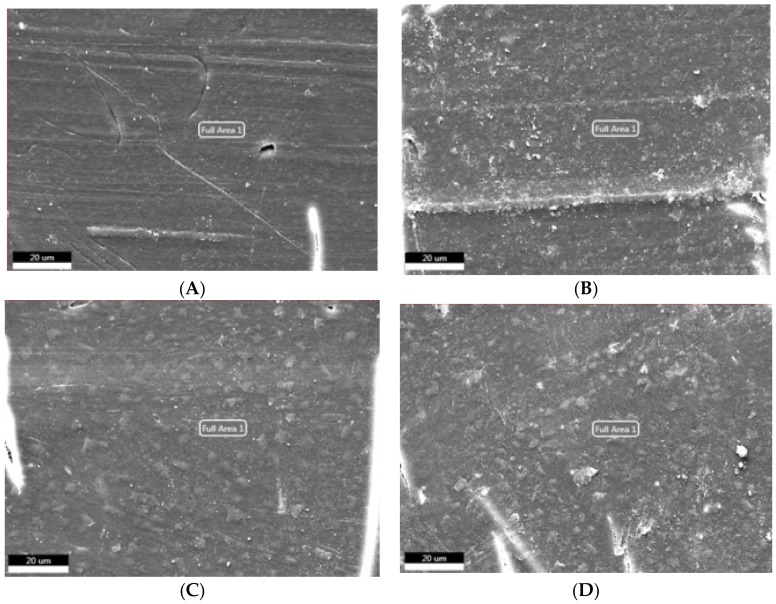
The selected area of EDAX on the samples’ surfaces: (**A**) unaged sample; (**B**) 20 d aged; (**C**) 40 d aged; and (**D**) 60 d aged.

To investigate the element variation of samples in thermal-oxidative ageing period, energy dispersive X-ray analysis (EDAX) is applied to analyse the element content on the surface of the composites. Figure 6 shows the photos of selected areas of EDAX by SEM, and the results of notable element contents (including C, O, and P) are listed in Table 2. It is observed that the content of oxygen remains at a relatively stable level in 0–40 d ageing, while is increases after 60 d of ageing, which is attributed to the long-term oxidation of the PBT matrix. It also can be clearly found that the content of phosphorus increases gradually from 20–60 d of ageing on the sample surface. We infer that the increase of phosphorus content is due to the diffusion of DOPO from the inner structure out to the composite surface through the oxygen and heat flow, which would affect the thermal properties and flame retardancy, as will be discussed in the next section.

### 3.4. Flammability and Thermal Stability

The LOI test is usually used to evaluate the fire resistance of polymer composites, especially to screen for the flame retardant formulation of materials [22,23]. Table 3 displays the LOI values of PBT/LGF/DOPO composites. The values of LOI decrease from 25.2% to 23.8%, while the ageing time just reaches 10 days, then increase gradually to 24.3% in the following period of thermal-oxidative ageing. In the early period of thermal-oxidative ageing, a certain extent of oxidation has occurred in the PBT matrix, which can generate some free radicals, like H–O·and H·. These free radicals cause the matrix to oxidize more easily in combustion, reflected by the lower value of LOI in 10 d ageing. With longer ageing time, the thermo-oxidative ageing can carbonize a part of the PBT matrix, which can be distinguished by the colour of the samples. The increase of LOI after 20 days of ageing are attributed to the aged carbonized parts of the composites which are much more difficult to burn than the original parts.

**Figure 7:**
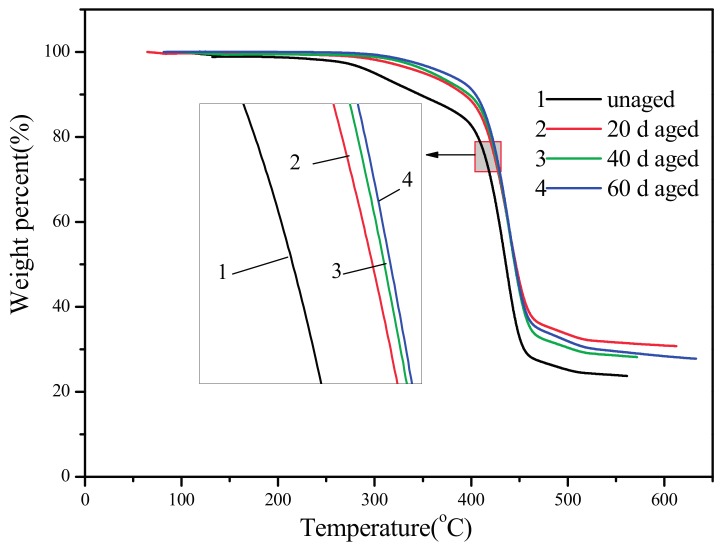
TGA curves of PBT/LGF/DOPO composites with different thermal-oxidation aging times.

Figure 7 shows the TGA curves of PBT/LGF/DOPO composites with different ageing times, and the corresponding data are listed in Table 4. It is observed that the curves show there is only one degradation stage for each aged sample and TGA curves move to higher temperatures with longer ageing time. For unaged composites, the initial degradation temperature (set as T_5%_) is 50 °C lower than that of aged ones, while T_max_ and the residue rate are also lower. All the data above indicate that the thermal-oxidative ageing gives the composites better thermal stability, which can be attributed to both the ageing procedure and the performance of the DOPO filler. In the course of thermal-oxidative ageing, DOPO can diffuse more easily with the assistance of heat flow and gather on the surfaces of the samples, which has been proved by the EDAX analysis above. The longer the ageing time, the greater the content of DOPO gathers on surface. In the course of thermal degradation, the P–H bond in DOPO firstly degrades into phosphoric acid, then reacts with –OH radicals generated by the PBT matrix, and the phosphorus-oxygen (P–O) free radicals which are obtained from DOPO can also capture plenty of hydrogen (H) and hydorgen-oxygen (H–O) free radicals generated by the PBT matrix during degradation. This carbonization effect occurs in some degree in the course of thermo-oxidative ageing, which makes the residue content higher than in unaged samples. These processes absorb heat, dehydrate the PBT matrix, and fix phosphorus into dense carbon layers on the sample’s surfaces, which can isolate the heat flow and gas to improve the thermal stability of the composites.

### 3.5. Dynamic Mechanical Properties

**Figure 8:**
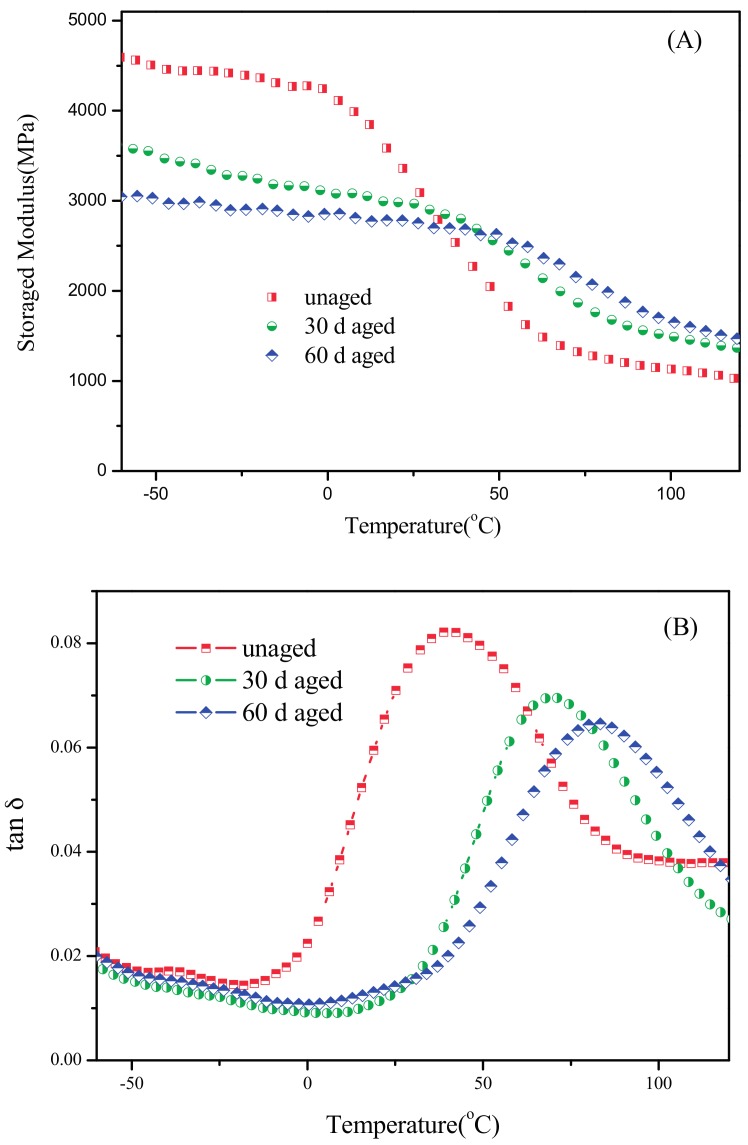
The DMA spectra of PBT/LGF/DOPO composites as a function of ageing time: (**A**) storage modulus; (**B**) tan δ.

DMA data for composites can provide the information about the glass transition temperatures of components to give a better observation of the phase structure and interphase mixing of the composites [24]. Plots of the storage modulus (E’) and loss factor (tan δ) as a function of temperature for PBT/LGF/DOPO composites with the frequency of 5 Hz are displayed in Figure 8A,B. In Figure 8A, the magnitude of the storage modulus of aged and unaged PBT/LGF/DOPO composites decrease approximately linearly as a function of temperature in the glassy region (lower than 0–20 °C). As shown in Figure 8B, there is only one peak on the curves of tan δ, which corresponds to α relaxation arising from the chain segmental motion of molecules, which is usually defined as the glass transition temperature (T_g_). Then the values of E’ in the glassy region (E’_g_) and the rubbery region (E’_r_) (measured at –60 °C and 120 °C, respectively), T_g_, and tan δ_max_ are shown in Table 5. The T_g_ shift to higher temperatures and the values of E_g_’ in the glass region and tan δ_max_ become lower with longer ageing time. Otherwise, the values of E_r_’ at 120 °C recover with longer ageing time, showing the same trend with the variation of mechanical properties which can be attributed to the embrittlement, as analysed above. The results of the increase of T_g_ from 0 d to 60 d is attributed to both the molecular chain crosslinking reaction in the primary stage of ageing and the partial carbonization caused by the reaction with DOPO in the later period, while the later one not only yellows the composites, improving the thermal properties, but also affects the dynamic mechanical properties. Then, the decrease in E_g_’ and tan δ_max_ are due to the molecular degradation over the whole period of ageing. The peak intensity (tan δ_max_) at T_g_ is considered to reflect the extent of the mobility of the macromolecular chain segments [25]. The lower value of tan δ_max_ implies that the mobility of molecular and damping properties are reduced after thermo-oxidative ageing. In fibre-reinforced polymer composites, there are several cases of damping decrease, like the variation in the interface properties due to the thermal stress and changes in the polymer conformation or morphology. The damping effects are relatively weak below T_g_. The widespread transition around T_g_ can be due to the short hard-segment units and could have also been related to an interface of the polymer weakly adsorbed on the surfaces of the filler of the glass fibre [25]. Obviously, even though both thermal embrittlement and oxidative embrittlement would make the chain relaxation much more difficult, because of the cross-linking of the molecule chains, the aggregation of weakness assembles in the amorphous regions to make the chain relaxation much more difficult. Add to this the effect of partial carbonization by DOPO, the composites show the embrittlement trend in the variation of global mechanical properties with long-term ageing, as discussed above in Section 3.1.

## 4. Conclusions

The influence of thermo-oxidative ageing on the static and dynamic mechanical properties, flammability and thermal stability, and morphology of LGF/PBT/DOPO composites at 120 °C for 0–60 days are investigated in this work. The experimental results indicate that the cracking and debonding, the crosslinking and variations of crystallinity, and the molecular degradation composites are responsible for the evolution of the static and dynamic properties and thermal properties in the ageing process. All of the mechanical strength decreases in the primary stage of ageing, while embrittlement occurs in the later period, and the crystallinity shows the same trend of decrease and recovery. The elongation at break of PBT/LGF/DOPO composites gradually decreases with increased ageing time. The morphology observation infers that the cracks and the heat damage start from the surface to penetrate the inner structure of materials and the interface debonding causes the mechanical properties to decrease. After more than 40 days of ageing, the variation of the LOI and TGA results indicate an obvious increase in the thermal stability for composites, which is due to the diffusion of DOPO and partial carbonization in composites, and it has also been proved by the EDAX method in the measurement of the content of phosphorus on the sample’s surface. The T_g_ shift to a higher temperature and storage modulus recover with longer ageing time at 120 °C in DMA results, also proving that degradation, crosslinking, and carbonization occurs in PBT/LGF/DOPO composites.

## Figures and Tables

**Table 1:** DSC results of PP/LGF/DOPO composites of different ageing times.

Ageing Time (Days)	T_m_/°C	ΔH_m_/(J/g)	X_c_/%
0	212.48	24.84	24.13
20	209.63	22.07	21.44
40	208.78	24.59	23.89
60	209.54	23.93	23.24

**Table 2:** The element weight content on the surface of unaged and aged PBT/LGF/DOPO samples.

Ageing Time (Days)	Element Weight (wt %)		
	C	O	P
unaged	30.44	51.95	4.64
20	29.34	50.97	5.05
40	32.24	50.51	5.91
60	31.27	54.24	7.29

**Table 3:** LOI of PBT/LGF/DOPO composites with different aging times.

Samples	LOI (%)
unaged	25.2
10 d	23.8
20 d	23.6
30 d	23.9
40 d	23.8
50 d	24.0
60d	24.3

**Table 4:** TGA data of PBT/LGF/DOPO composites with different thermal-oxidation aging times.

Ageing Time (Days)	T_5%_ (°C)	T_max_ (°C)	Residue Rate (%)
0	300.07	435.79	23.73
20	351.27	439.24	30.76
40	360.07	439.74	28.17
60	373.01	439.51	27.80

**Table 5:** Values of E’, T_g_, and tan δ_max_ of PBT/LGF/DOPO composites with different ageing times.

Ageing Time (Days)	E’_g_ (MPa)	E’_r_ (MPa)	T_g_ (°C)	Tan δ_max_
0	4592	1018	40.58	0.0822
30	3599	1362	69.43	0.0697
60	3044	1459	81.65	0.0645
